# Genetic analysis of chikungunya viruses imported to mainland China in 2008

**DOI:** 10.1186/1743-422X-7-8

**Published:** 2010-01-18

**Authors:** Kui Zheng, Jiandong Li, Quanfu Zhang, Mifang Liang, Chuan Li, Miao Lin, Jicheng Huang, Hua Li, Dapeng Xiang, Ninlan Wang, Ye Hong, Li Huang, Xiaobo Li, Deguan Pan, Wei Song, Jun Dai, Boxuan Guo, Dexin Li

**Affiliations:** 1 Guangdong Inspection and Quarantine Technology Center, Guangzhou, China; 2 State Key Laboratory for Molecular Virology and Genetic Engineering, Institute for Viral Disease Control and Prevention, China CDC, 100 Yingxinjie, Xuanwu District, Beijing 100052, China; 3 State Key Laboratory for Infectious Disease Control and Prevention, 100 Yingxinjie, Xuanwu District, Beijing 100052, China; 4 Guangdong Entry-exit Inspection and Quarantine Bureau, Guangzhou, China; 5 Guangzhou Baiyun Airport Entry-exit Inspectional and Quarantine Bureau, China

## Abstract

**Background:**

Chikungunya virus (CHIKV) has caused large outbreaks worldwide in recent years, especially on the islands of the Indian Ocean and India. The virus is transmitted by mosquitoes (*Aedes aegypti*), which are widespread in China, with an especially high population density in southern China. Analyses of full-length viral sequences revealed the acquisition of a single adaptive mutation providing a selective advantage for the transmission of CHIKV by this species. No outbreaks due to the local transmission of CHIKV have been reported in China, and no cases of importation were detected on mainland China before 2008. We followed the spread of imported CHIKV in southern China and analyzed the genetic character of the detected viruses to evaluate their potential for evolution.

**Results:**

The importation of CHIKV to mainland China was first detected in 2008. The genomic sequences of four of the imported viruses were identified, and phylogenetic analysis demonstrated that the sequences were clustered in the Indian Ocean group; however, seven amino acid changes were detected in the nonstructural protein-coding region, and five amino acid changes were noted in the structural protein-coding regions. In particular, a novel substitution in E2 was detected (K252Q), which may impact the neurovirulence of CHIKV. The adaptive mutation A226V in E1 was observed in two imported cases of chikungunya disease.

**Conclusions:**

Laboratory-confirmed CHIKV infections among travelers visiting China in 2008 were presented, new mutations in the viral nucleic acids and proteins may represent adaptive mutations for human or mosquito hosts.

## Background

Chikungunya virus (CHIKV) is a mosquito-transmitted alphavirus belonging to the family Togaviridae, with an envelope and single-stranded positive-sense RNA genome. The genome, which is 11 to 12 kb in length, is organized with nonstructural proteins (nsP1-4) at the 5'-end and structural proteins (Capsid-E3-E2-6k-E1) at the 3'-end [1]. CHIKV is responsible for an acute infection of abrupt onset, characterized by a high fever, arthralgia, myalgia, headache, and rash [2]. The virus is transmitted mainly from human to human by the bite of the *Aedes *mosquito, primarily *Aedes aegypti*. A large outbreak of chikungunya disease (CHIK) occurred in India in 2005-06, with more than 1 million cases reported [3]. It is believed that the outbreak originated in Kenya during 2004, was followed by outbreaks on islands in the southwestern Indian Ocean in early 2005 [4]. Outbreaks continued to be reported in many other countries, including Gabon, India, Indonesia, Italy, Malaysia, and Sri Lanka, in 2007, and Singapore and Sri Lanka in 2008. Most of these outbreaks were attributed to variants of the central/east African genotype of CHIKV [5], but in Malaysia, co-circulating genotypes of CHIKV (Asian and central/east African) were reported [6,7].

RNA viruses are usually highly genetically diverse, and their genomes contain signs of past and present variation and mobility. High mutation rates and quasispecies dynamics have conferred on them significant adaptive potential. Genetic analyses of viral genomic sequences conducted over relatively short times during an outbreak can be used to distinguish between different strains of a virus. Such data are helpful in understanding the evolutionary potential of a virus and mechanisms underlying the development of a disease outbreak; moreover, they may be used in the development of new strategies for viral disease prevention and control. The outbreaks in the Indian Ocean, which were of unprecedented magnitude, were partially due to a mutation in the E1 protein of CHIKV (A226V), which helped in the adaptation of the virus to *Aedes albopictus *[3,8].

*Aedes albopictus *is widespread in China, with an especially high population density in southern China. No outbreaks due to the local transmission of CHIKV have been reported in China, and no importation of cases was detected in mainland China before 2008. Humans are the major source, or reservoir, of CHIKV for mosquitoes; the mosquito transmits the disease by biting an infected person and then biting someone else. The introduction and spread of CHIKV outbreaks in China is a potential threat. Therefore, it is important to strengthen the surveillance of CHIKV and prevent the localization of imported CHIKV. Here, we report five imported cases of CHIK from Sri Lanka and Malaysia during March, October, and December of 2008, respectively. Whole-genome analyses of four CHIKV were performed, and unique nucleic acid and amino acid changes, as compared to CHIKVs isolated during different years, from different geographical regions, and from different clades, were detected.

## Materials and methods

### Patients and Clinical Specimens

Patients with a fever (up to 37.8°C) were identified from three travel groups visiting or returning to China from March to December in 2008. Serum samples from the patients and 30 close contacts were collected for dengue and CHIKV testing (Table 1). All work with potentially infectious material was performed in a biosafety level 2 or 3 containment facility.

**Table 1:** Patients and serum sample collection (2008)

	Patient/contacts	Visiting date 2008	Country of origin *	Illness onset date 2008	Collection date 2008	Nr.	Sign and syndrome			
							
							Fever (°C)	Conj.	Arth.	Bleed.
I	1	4 Mar.	LK	4 Mar.	4 Mar.	1	38.2	-	-	-
	2	4 Mar.	LK	12 Mar.	16 Mar.	1	38.0	-	+	-
	Contacts	4 Mar.	LK	-.	16 Mar.	18	-	-	-	-
II	3	3 Oct.	MY	3 Oct.	3 Oct.	1	38.6	+	-	+
	4	3 Oct.	MY	8 Oct.	10 Oct.	1	37.8	-	+	-
	Contacts	3 Oct.	MY	-	10 Oct.	7	-	-	-	-
	Contacts	-	CN	-	10 Oct.	3	-	-	-	-
III	5	27 Dec.	MY	27 Dec.	28 Dec.	1	39.0	+	-	-
	Contacts	27 Dec.	MY	-	29 Dec.	2	≥37.5	-	-	-

*LK, Sri Lanka; MY, Malaysia; CN, China; Conj., Conjunctivitis; Arth., Arthralgia; Bleed., Bleeding

### IgM and IgG Detection in Serum

Sera from the patients and close contacts were tested for the presence of IgM or IgG antibodies against dengue or CHIKV. Dengue virus-specific antibodies were detected using a Panbio MacELISA kit for IgM and indirect ELISA kit for IgG according to the manufacturer's instructions. CHIKV-specific antibodies were detected using an indirect immunofluorescence test (IIFT) (EUROIMMUN, Lübeck, Germany). In brief, the samples were diluted 1:10-1:80, and 20 μL were applied to the reaction fields of the BIOCHIPs, which were then incubated for 1 h. For the detection of IgM and IgG, rheumatic factor was pre-adsorbed with EUROSORB reagent (EUROIMMUN). For antibody detection, anti-human IgG or IgM antibodies labeled with fluorescein isothiocyanate (FITC) were used. The results were evaluated by fluorescence microscopy; titers ≥1:10 were considered positive.

### Diagnostic RT-PCR

The extraction of viral RNA from the CHIKV isolates or human sera was achieved using a QIAamp Viral Minikit (Qiagen, Hilden, Germany) according to the manufacturer's recommended procedures. The partial sequence of the gene encoding E2 from CHIKV was amplified by nested PCR using a previously described method [9] with a Qiagen OneStep RT-PCR Kit. Products of the expected sizes (427 and 172 bp) were obtained by outer and inner PCR, respectively. The outer product was sequenced using an automated ABI 3100 Genetic Analyzer with the PCR primers, without further cloning. Subsequently, the obtained sequence was blasted against GenBank to verify the amplified fragments.

### CHIKV Isolation

A CHIKV stock was isolated by placing 10 μL of patient serum (SD08Pan) at a dilution of 1:10 onto a confluent monolayer of Vero cells in a 24-well plate. The virus was allowed to absorb for 1 h at 37°C, after which 1 mL of Dulbecco's modified Eagle's medium supplemented with 2% fetal calf serum (FCS) and antibiotics was added and the plate was incubated at 37°C under 5% CO_2_. The cultures were checked daily for cytopathic effects (CPEs). At approximately 80% CPE, the cells and supernatants were harvested (day 5). The isolates were identified as CHIKV by genomic sequencing via RT-PCR.

### Nucleotide Sequencing and Sequence Analysis

To obtain the complete sequence of the CHIKV genome, primers were designed based on an alignment of all of the CHIKV genomic sequences published in GenBank (available upon request). Viral RNA was obtained directly from patient serum or a viral isolate after one passage in Vero cells. Reverse transcription was performed with a SuperScript III cDNA Synthesis Kit (Invitrogen, Carlsbad, CA, USA). Amplification was achieved using a Platinum^® ^Taq DNA Polymerase High Fidelity Kit (Invitrogen). The products were purified using 1.2% agarose gels with Qiaquick Spin Columns (Qiagen). Sequencing reactions were run using the BigDye Terminator v1.1 Cycle Sequencing Kit (Applied Biosystems, Foster City, CA, USA) and purified by ethanol precipitation. Sequence chromatograms were obtained on ABI3100 automated sequence analyzers (Applied Biosystems). All amplicons were sequenced twice on each strand. Sequences were assembled with the DNA-Star software package and compared with ClustalW 1.83 to previously published CHIKV sequences in GenBank. Neighbor-joining trees were constructed using MEGA version 4.0 [10]. Evolutionary distances were computed using the Maximum Composite Likelihood method [11] and are given as the number of base substitutions per site. All positions containing gaps and missing data were eliminated from the dataset. There were a total of 11594 positions in the final dataset.

## Results

### Case discovery and sample collection

On March 4, 2008, a group of 20 Chinese men returned to China through Guangzhou Airport via Malaysia after about six months in Sri Lanka. One of the men (41 years old) presented with a flu-like syndrome and a fever of 38.2°C. On March 12^th^, a second man (50 years old) developed a fever of up to 38°C with joint pain in the knees. In October of 2008, a group of nine people from Malaysia visited Guangdong Province through Guangzhou Airport, one of whom (a 26-year-old man) appeared feverish at customs. Physical examination revealed a flu-like syndrome with a fever of 38.6°C, hemorrhagic petechia around the body, and no joint pain. Five days later, the father of the patient (a 63-year-old man) presented with a fever of 37.8°C and pain in the joints of his knees and fingers. In December of 2008, an eight-person family group came to China from Malaysia through Guangzhou Airport. A 44-year-old male in the group was found to have a fever of 39°C and conjunctivitis. The three patients imported from Malaysia during October to December of 2008 had lived in Malaysia for a long time with no recent outside travel history or contact with infectious disease patients. A general description of each clinical case and of the serum samples collected from the above five patients and 30 close contacts is given in Table 1; the cases were classified as suspected cases of dengue or CHIKV infection.

### Laboratory tests and diagnosis

The serum samples from the suspected patients and close contacts were tested by ELSIA, IIFT, and RT-PCR for dengue and CHIKV. Evidence of CHIKV infection was found in all five patients classified as a suspected case (Table 2). The serum in case 1 (FD080008; returning from Sri Lanka) was weakly positive for dengue IgM and IgG antibodies, suggesting a dengue viral infection; however, cross reaction could not be excluded. Four of the five cases appeared to be CHIKV IgM-positive. Only one serum sample, SD08Pan, was CHIKV IgG-positive. RT-PCR revealed that the five cases were infected with CHIKV. Nucleotide sequence analysis in each case amplified a 427-bp fragment, showing that the viral strains belonged to a group of viruses identified recently in the Indian Ocean (data not shown). Of the 30 samples from close contacts, no evidence of dengue and/or CHIKV infection was detected.

**Table 2:** Diagnostic test results for five imported cases in China (2008)

Case	Dengue			CHIKV			Illness onset date 2008	Days from onset to collection	Genome sequencing
				
	IgM	IgG	PCR	IgM	IgG	PCR			
1	+	+	-	+	-	+	4 Mar.	1	FD080008
2	-	-	-	+	+	+	12 Mar.	4	SD08pan
3	-	-	-	+	-	+	3 Oct.	1	FD080178
4	-	-	-	-	-	+	8 Oct.	2	N.A.*
5	-	-	-	+	-	+	27 Dec.	1	FD080231

*N.A., not available (whole-genome sequence analysis was not performed)

### Virus isolation

Three days after cell culture inoculation using serum from patient 2, a CPE was observed, which was transferable following cell-free passage of the supernatant to fresh Vero cells. RNA was extracted from the supernatant of the first passage and analyzed by RT-PCR and nucleotide sequencing. The supernatant was found to contain CHIKV (referred to as SD08Pan).

### Genetic analysis

The nearly complete genomic sequences of four imported CHIKVs (FD080008 and SD08Pan from Sri Lanka and FD080178 and FD080231 from Malaysia) were determined [GenBank: GU199350-GU199353]. The genomic sequences of FD080008, FD080178, and FD080231 were obtained directly from the sera of patients in the acute phase; the sequence of SD08Pan was obtained from viral isolates in the first passage. Using these whole-genome sequences, we sought to detect genetic variation between our CHIKVs and those isolated during a large outbreak in the Indian Ocean.

**Figure 1:**
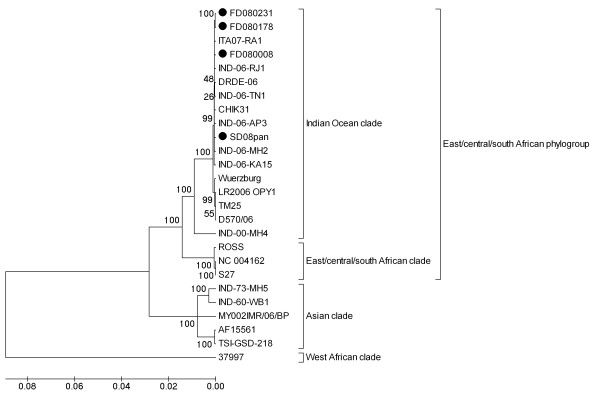
**Phylogeny of four CHIKVs imported into China during 2008**. The whole-genome sequences obtained in this study are indicated by black dots. Neighbor-joining trees were constructed using MEGA version 4.0 [10]; evolutionary distances were computed using the Maximum Composite Likelihood method [11] and are given as the number of base substitutions per site. All positions containing gaps and missing data were eliminated from the dataset. There were a total of 11594 positions in the final dataset.

We compared an 11677-bp region from our viruses with 22 other complete genomic sequences taken from CHIKVs isolated during different years. Phylogenetic analysis clearly demonstrated that the four viral sequences belonged to the homogeneous Indian Ocean clade (Fig. 1). Viruses FD080178 and FD080231 (imported from Malaysia) were less related to the Asian isolates, which were recently reported to be the cause of a reemergence of endemic CHIK in Malaysia [6]. Changes in sequence, compared to 22 published CHIKV whole-genome sequences from GenBank, were detected in FD080008 (8 bp), SD08Pan (12 bp), FD080178 (11 bp), and FD080231 (7 bp) (Additional file 1 and 2). These changes produced specific amino acid changes in FD08008 (nsP3-M394I, E2-R178H, and 6K-V31I), SD08Pan (nsP1-Q120R, nsP2-G577R, nsP2-N632S, and nsP3- D372N), FD080178 (nsP2-L539S, nsP4-P181S, C-T8A, E2-K252Q, and E1-A306V), and FD080231 (nsP2-L539S, C-T8A, and E2-K252Q) (Table 3). Notably, nsP2 (L539S), C (T8A), and E2 (K252Q) appeared in both isolates imported from Malaysia (FD080178 and FD080231). In addition to these changes, there were a total of 19 silent nucleotide substitutions in the four imported viruses (Additional file 2). E1 A226V was observed in FD080178 and FD080231, while the other two Sri Lankan viruses (FD080008 and SD08Pan) had E1-226A.

**Table 3:** Unique amino acid changes observed in four imported CHIKVs compared to 22 published strains^a^

	Non-structural proteins							Structural proteins				
		
	nsP1	nsP2	nsP2	nsP2	nsP3	nsP3	nsP4	C	E2	E2	6K	E1
Polypeptide position*	120	1074	1112	1167	1705	1727	2044	8	503	577	779	1125
Protein position*	120	539	577	632	372	394	181	8	178	252	31	316
FD080008	Q	L	G	N	D	M→I	P	T	R→H	K	V→I	A
SD-08-pan	Q→R	L	G→R	N→S	D→N	M	P	T	R	K	V	A
FD080178	Q	L→S	G	N	D	M	P→S	T→A	R	K→Q	V	A→V
FD080231	Q	L→S	G	N	D	M	P	T→A	R	K→Q	V	A

^a^Twenty-two CHIKV whole-genome sequences were selected for comparison with the imported CHIKVs, including three east/central/south African strains: S27 [GenBank:AF369024], NC004162 [GenBank:NC_004162], and ROSS [GenBank:AF490259]; 13 Indian Ocean strains: IND-00-MH4 [GenBank:EF027139], IND-06-TN1 [GenBank:EF027138], CHIK31 [GenBank:EU564335], TM25 [GenBank:EU564334], DRDE-06 [GenBank:EF210157], ITA07-RA1 [GenBank:EU244823], Wuerzburg [GenBank:EU037962], D570/06 [GenBank:EF012359], IND-06-RJ1 [GenBank:EF027137], IND-06-MH2 [GenBank:EF027136], IND-06-KA15 [GenBank:EF027135], IND-06-AP3 [GenBank:EF027134], and LR2006 OPY1 [GenBank:DQ443544]; five Asian strains: IND-60-WB1 [GenBank:EF027140], IND-73-MH5 [GenBank:EF027141], AF 15561 [GenBank:EF452493], TSI-GSD-218 [GenBank:L37661], and MY002IMR/06/BP [GenBank:EU703759]; and one West African strain: 37997 [GenBank:AY726732]. *S27 reference numbering.

## Discussion

Although the large epidemic of CHIK across the islands of the Indian Ocean is now in decline, outbreaks of the Indian Ocean strains were reported in many other countries, and opportunities for the introduction of CHIKV to China were not limited.

In this study, for the first five cases of CHIK detected in China in 2008, the possible transmission of the virus carried by the travelers was monitored. Although, of the five imported cases, secondary cases were detected among the travelers from groups I and II at eight and five days after the onset of the first case in the group, respectively, we believe that the infections originated in Sri Lanka and Malaysia, respectively, rather than in China, as the disease has an incubation period of about three to twelve days [2] and no other local close contacts were found to be infected. For the two close contacts of the cases from group III (from Malaysia) who presented with a mild fever and were suspected to have a CHIKV infection, laboratory analysis did not support the suspicion and additional samples were not collected because the individuals left China soon after the onset of the first case in the travel group.

The amino acid differences detected among the imported CHIKVs might be related to their biological or pathogenic characteristics. To examine the genetic variation among the imported CHIKVs, a whole-genome analysis was performed based on 22 published CHIKV sequences. To prevent viral genome mutations caused by cell passage, we obtained our whole-genome sequences directly from clinical serum samples (FD080008, FD080178, and FD080231) or viral isolates (SD08P) and passaged in Vero cells only once.

Nonstructural proteins (nsPs) are involved in viral replication [12,13], and studies of other alphaviruses have suggested a strong effect for point mutations or deletions in nsP1 and nsP3 on the neurovirulence of Sindbis virus [14]. Strain SD08Pan, which was passaged only once in Vero cells, showed four amino acid changes in its nsPs (nsP1-Q120R, nsP2-G577R, nsP2-N632S, and nsP3- D372N); in comparison, the other sequences identified directly from patient serum showed only one or two amino acid changes (nsP3-M394I in FD080008, nsP2-L539S and nsP4-P181S in FD080178, and nsP2-L539S in FD080231). These may indicate the evolutionary potential of the virus.

In alphaviruses, structural proteins E2 and E1 occur as a closely associated heterodimer on the surface of the virion, with E2 projecting outward and over E1, covering the E1 fusion loops [15,16]. The fusion of flaviviruses and alphaviruses with host cell membranes requires activation by proteolytic cleavage and a reduced pH as a trigger for conformational change. When alphavirus particles are exposed to a low pH, protein packing in the icosahedral surface lattice changes substantially [17,18]. The E1-E2 heterodimers dissociate and E1 trimerizes, causing the E2 subunits to move away, permitting clustering of E1 to initiate fusion. Mutations around a hydrophobic pocket could alter the threshold pH for flavivirus fusion clustering [19]. Residue 243 in E2 is likely to be the major determinant of neurovirulence within the structural proteins [14]; near this position, both FD080178 and FD080231 showed a unique change (E2 K252Q) in which a strongly basic amino acid was changed to a neutral one. This might alter the fusion ability of FD080178 and FD080231. Other changes in structural proteins (e.g., E2-R178H and 6K-V31I in FD08008 and C T8A and E1-A306V in FD080178) were detected; however, the effect on viral maturation and pathogenesis is unclear.

In conclusion, the data reported here confirm that CHIKV was imported into China, although transmission from the travelers' carrying CHIKV did not occur. In addition, the nucleic acid and amino acid changes described here may indicate recent evolution in the ability of the virus to cause an infection. Thus, clinician attention and public health laboratory surveillance should be enhanced in China.

## Competing interests

The authors declare that they have no competing interests.

## Authors' contributions

ZK participated in sample collection, detection, and whole-genome sequencing. LJ participated in sample collection, detection, whole-genome sequencing, genetic analysis, and the drafting of the manuscript. ZQ participated in sample collection, detection, and viral isolation. LM participated in the design of the study and editing of the manuscript. LC participated in sample collection, detection, and viral isolation. LM, WN, HY, HL, LX, PD, SW, JD, and GB participated in sample collection and detection. HJ provided reagents and participated in the design of the study, sample collection, and detection. LH and XD participated in the design and coordination of the study. LD provided reagents and participated in the design and coordination of the study, as well as in the analysis of the data and drafting and editing of the manuscript. All authors read and approved the final manuscript.

## Supplementary Material

Additional File 1 **Table S1**. Unique nucleic acid and related amino acid changes detected in the four imported CHIKVs. Click here for file

Additional File 2 **Table S2**. Relevant synonymous changes identified in the imported Indian Ocean virus versus a selection of 22 CHIKV sequences. Click here for file
